# High IRF8 expression correlates with CD8 T cell infiltration and is a predictive biomarker of therapy response in ER-negative breast cancer

**DOI:** 10.1186/s13058-021-01418-7

**Published:** 2021-03-25

**Authors:** Gerardo Gatti, Courtney Betts, Darío Rocha, Maribel Nicola, Verónica Grupe, Cecilia Ditada, Nicolas G. Nuñez, Emiliano Roselli, Paula Araya, Jeremías Dutto, Lucia Boffelli, Elmer Fernández, Lisa M. Coussens, Mariana Maccioni

**Affiliations:** 1 Laboratorio de Investigación en Cáncer, Fundación para el progreso de la Medicina, X5000EMS Córdoba, Argentina; 2 Consejo Nacional de Investigaciones Científicas y Técnicas (CONICET), Buenos Aires, Argentina; 3 Department of Cell, Developmental & Cancer Biology, Knight Cancer Institute, Oregon Health & Science University, Portland, OR USA; 4 Facultad de Ciencias Exactas, Físicas y Naturales, Universidad Nacional de Córdoba, Córdoba, Argentina; 5 Institute of Experimental Immunology, University of Zurich, Zurich, Switzerland; 6 Departamento de Bioquímica Clínica, Centro de Investigaciones en Bioquímica Clínica e Inmunología (CIBICI), Consejo Nacional de Investigaciones Científicas y Técnicas (CONICET), Facultad de Ciencias Químicas, Universidad Nacional de Córdoba, 5000 Córdoba, Argentina; 7 CIDIE-CONICET, Universidad Católica de Córdoba, Córdoba, Argentina

**Keywords:** Breast cancer, IRF8, DNA methylation, Predictive marker, Tumor-infiltrate

## Abstract

**Background:**

Characterization of breast cancer (BC) through the determination of conventional markers such as ER, PR, HER2, and Ki67 has been useful as a predictive and therapeutic tool. Also, assessment of tumor-infiltrating lymphocytes has been proposed as an important prognostic aspect to be considered in certain BC subtypes. However, there is still a need to identify additional biomarkers that could add precision in distinguishing therapeutic response of individual patients. To this end, we focused in the expression of interferon regulatory factor 8 (IRF8) in BC cells. IRF8 is a transcription factor which plays a well-determined role in myeloid cells and that seems to have multiple antitumoral roles: it has tumor suppressor functions; it acts downstream IFN/STAT1, required for the success of some therapeutic regimes, and its expression in neoplastic cells seems to depend on a cross talk between the immune contexture and the tumor cells.

The goal of the present study was to examine the relationship between IRF8 with the therapeutic response and the immune contexture in BC, since its clinical significance in this type of cancer has not been thoroughly addressed.

**Methods:**

We identified the relationship between IRF8 expression and the clinical outcome of BC patients and validated IRF8 as predictive biomarker by using public databases and then performed in silico analysis. To correlate the expression of IRF8 with the immune infiltrate in BC samples, we performed quantitative multiplex immunohistochemistry.

**Results:**

IRF8 expression can precisely predict the complete pathological response to monoclonal antibody therapy or to select combinations of chemotherapy such as FAC (fluorouracil, adriamycin, and cytoxan) in ER-negative BC subtypes. Analysis of immune cell infiltration indicates there is a strong correlation between activated and effector CD8^+^ T cell infiltration and tumoral IRF8 expression.

**Conclusions:**

We propose IRF8 expression as a potent biomarker not only for prognosis, but also for predicting therapy response in ER-negative BC phenotypes. Its expression in neoplastic cells also correlates with CD8^+^ T cell activation and infiltration. Therefore, our results justify new efforts towards understanding mechanisms regulating IRF8 expression and how they can be therapeutically manipulated.

**Supplementary Information:**

The online version contains supplementary material available at 10.1186/s13058-021-01418-7.

## Background

Interferon regulatory factor 8 (IRF8) is a transcription factor of the interferon regulatory factor family that plays an essential role in the development and maturation of myeloid cells and in the expression of genes involved in antigen capture, processing, and presentation as well as in the activation of these cells in response to IFN-γ, IFN-β, and pro-inflammatory stimuli [1–6].

IRF8 has also been found to be expressed and functional in non-hematopoietic cells such as epithelial cells [7, 8]. Studies of IRF8 mRNA levels in normal human colon and colorectal carcinoma (CRC) revealed that IRF8 is downregulated in tumor tissues as compared to non-malignant counterpart tissues. Moreover, analysis of The Cancer Genome Atlas (TCGA) datasets in CRC revealed that the IRF8 promoter DNA is more methylated in tumors than in normal colon tissue, indicating that its expression is in part controlled by epigenetic mechanisms [9]. Similar data have been reported for nasopharyngeal carcinoma, esophageal cancer, breast cancer, cervical cancer, and especially in lung carcinoma [10, 11].

In a model of inflammation-induced spontaneous colon cancer, mice with IRF8 deficiency specifically in colon epithelial cells contained an increased percentage of Ki67^+^ cells in the stem cell zones of the crypt as compared to wild type mice and developed more tumor nodules when subjected to azoxymethane-DSS cycles [9]. In lung cancer cells, IRF8 negatively regulates Akt phosphorylation, inducing cellular senescence [12]. In addition, inhibition of IRF8 expression levels has been found to increase tumor growth in lung cancer xenografts, indicating a role for IRF8 in progression of late-stage lung cancers [12]. Thus, it has been proposed that IRF8 could be acting as a tumor suppressor via mechanisms that need to be better elucidated. Accordingly, IRF8 has been found to act as tumor suppressor in other solid tumors and hematopoietic malignancies [13–15].

Less is known regarding the role of IRF8 in BC cells, but reports are consistent with a tumor suppressive role. For example, the IRF8 promoter appeared severely hypermethylated in metastatic BC patients [16]. Induced IRF8 expression in BC cell lines in vitro suppressed cell proliferation, colony formation, and cell migration and invasion and induced apoptosis by enhancing the pro-apoptotic effect of IFN-γ and suppressing β-catenin signaling [17]. Also, consistent with the role of IRF8 as a tumor suppressor, high expression of IRF8 has been significantly associated with longer overall survival in ER-negative BC [17].

Since IRF8 is downstream IFN/STAT1 signaling and there are certain therapeutic regimes that require this signaling axis for its efficacy, we investigated if its expression can predict therapeutic response. In this study herein, we support utilization of IRF8 expression as a potent biomarker not only for prognosis, but also for predicting therapeutic response in ER-negative BC subtypes (HER2+ and TNBC). In particular, we report that IRF8 expression predicts the complete pathological response to monoclonal antibody therapy (trastuzumab) or to certain combinations of chemotherapy such as FAC (fluorouracil, adriamycin, and cytoxan) in these BC subtypes. Moreover, analysis of immune contexture of tumor tissues indicates a strong correlation between activated and effector CD8^+^ T cell infiltration and tumoral IRF8 expression.

Our results raise new questions regarding the cross-talk between immune infiltrates and IRF8 expression in neoplastic cells and demands new efforts in studies aiming to understand how IRF8 expression levels are regulated and how they can be therapeutically manipulated.

## Material and methods

### Tissue microarray and immunohistochemistry

To examine IRF8 expression we performed immunohistochemistry on a BC commercial tissue microarray (TMA, US Biomax, USA, breast cancer molecular subtype was not provided). TMA was deparaffinized in xylene and rehydrated using graded alcohols, and antigen retrieval was performed using citrate buffer (30 min, 95 °C), followed by blocking and subsequent incubation with IRF8 antibody 1/1000 (Rabbit Anti-IRF8 monoclonal antibody [EPR20441] #ab207418, Abcam). The UltraView Universal diaminobenzidina tetrachloride (DAB) detection kit was used to detect protein expression. Slides were counterstained with Hematoxylin. IRF8 staining patterns were evaluated and scored based on intensity and percentage of positive cells as previously described [18]. The score is designated as 0 when no tumor cells stain, 1+ when 10–20% of cells stain (weak), 2+ when 20–50% of cells stain (moderate), and 3+ when > 50% of cells stain (strong). The immunohistochemistry scoring was performed by two observers that were blinded and the degree of agreement was good. Tumors were then stratified as IRF8 low (< 2 score) or IRF8 high (≥ 2 score).

For analysis of the immune infiltrates, we performed quantitative multiplex immunohistochemistry as previously described [19] on a formalin-fixed and paraffin embedded (FFPE) section of a homemade TMA, generated using ER-negative breast tumor tissue blocks (24 samples). Tumor samples were stained with anti-CD45 antibody and selection of tumor areas was made by choosing the regions with the highest quantity of CD45^+^ cells. Sections were matched to their corresponding paraffin blocks (donor blocks), and two tumor cores with a diameter of 2 mm were punched from tumor regions of each donor block and precisely arrayed into a new recipient paraffin block (TMA block) using the Galileo Tissue MicroArrayer CK 4500 (Transgenomic). Five-micrometer-thick slices were cut from the TMA FFPE blocks and transferred to glass histology slides which were processed, stained, and analyzed as previously described [19]. Briefly, chromogenic sequential IHC was conducted with 5 mm of FFPE tissue sections. Following deparaffinization, slides were stained by hematoxylin (S3301, Dako) for 1 min, followed by whole tissue scanning using Aperio ImageScope AT (Leica Biosystems). Slides were subjected to endogenous peroxidase blocking followed by heat-mediated antigen retrieval. Then, sequential IHC consisting of iterative cycles of staining, scanning, and antibody and chromogen stripping was performed as described in Tsujikawa et al. [19]. The digital image workflow encompasses three steps: image preprocessing, visualization, and quantitative image analysis. In image preprocessing, iteratively digitized images were coregistered so that cell features overlap down to a single-pixel level, using Matlab software. Visualization was performed via conversion of coregistered images to pseudo-colored single-marker images in ImageJ v.1.48 [20] and ImageScope (Leica Biosystems). In quantitative image assessment, single cell segmentation and quantification of staining intensity were performed using a CellProfiler v.2.1.1 pipeline, CellID_FlowCyt-6.9.15. cpproj (available under GPLv2 at https://github.com/multiplexIHC/cppipe). All pixel intensity and shape-size measurements were saved to a file format compatible with flow and image cytometry data analysis software, FCS Express 5 Image Cytometry v.5.01.0029 (De Novo Software). Expression of IRF8 was also corroborated in the custom TMA and tumors were stratified as IRF8 low (< 2 score) or IRF8 high (≥ 2 score). Tonsil tissue was used as positive control.

### Cell lines

MDA-MD231 (MDA231) and MCF7 cells were grown in DMEM containing 10% heat-inactivated FBS (Biowest), 100 IU/ml penicillin, 100 μg/ml streptomycin, 2.0 mM GlutaMAX (all from Thermo Fisher Scientific). All cell lines were tested as mycoplasma-negative by PCR.

### Sodium bisulfite treatment and MS-PCR analysis

Genomic DNA was purified using DNeasy Tissue kit (Qiagen) according to the manufacturer’s instructions. Sodium bisulfite treatment of genomic DNA was carried out using EZ DNA Methylation-Lightning Kit (Zymo Research) according to the manufacturer’s instructions. MS-PCR was carried out as previously described [10].

### Analyses using online databases

The MethHC database tool (http://methhc.mbc.nctu.edu.tw/) [21] was used to analyze the correlation of IRF8 methylation and gene expression in breast tumor samples compared to normal breast tissue samples. The relationships between IRF8 expression and BC patient overall survival and relapse free survival were analyzed using the Kaplan-Meier plotter data base (http://kmplot.com/analysis/) [22]. To validate IRF8 as predictive biomarker, we used the ROCplot website (http://www.rocplot.org/) [23]. The patients were assigned to two cohorts (responder and nonresponder) based on their clinical characteristics. Patients with neoadjuvant chemotherapy were classified according to pathological response as published by the authors. In this, instead of four cohorts (progressive disease, stable disease, partial response and complete response), all patients were assigned into two cohorts, including those where no residual histological evidence of the tumor remains after chemotherapy (responders) and all other patients with residual tumor tissue (nonresponders) [23]. Patient samples were grouped based on the expression of IRF8 (Jetset best probeset) using the median cutoff value.

### TCGA data analysis and MIXTURE

The results reported here are partially based on data generated by TCGA Research Network: https://www.cancer.gov/tcga. BC expression data was downloaded as RNA-seq rsem raw counts and TMM (https://genomebiology.biomedcentral.com/articles/10.1186/gb-2010-11-3-r25) normalized. BC survival data was truncated at 10 years follow-up time. Survival analyses cutoff values were roughly optimized by studying quantile cutoffs from 0.2 to 0.8 by 0.1 and choosing the cutoff with lowest *p* value. MIXTURE deconvolution method was used to estimate tumor immune infiltration from expression data. To study CD8^+^ T cells abundance in relation to IRF8 expression, IRF8 expression was categorized as high or low respective to the median IRF8 expression value. Isobaric tags for relative and absolute quantification (iTRAQ)-based mass spectrometry data from TCGA was used to interrogate protein expression in primary BC samples.

### Statistical analysis

Data handling, analysis, and graphic representation (all shown as mean ± SEM) were performed using Prism 7.0 (GraphPad Software), R statistical software (https://www.r-bloggers.com/its-easy-to-cite-and-reference-r/) and Survival package (https://cran.r-project.org/web/packages/survival/citation.html). For multiple comparison, two-way analyses of variance (ANOVA) with Sidak’s post-test were performed. For the comparison between two groups, two-tailed unpaired Student *T* test was performed. A *p* < 0.05 was considered statistically significant (*p < 0.05, **p < 0.01, ***p < 0.001). Comparisons for the abundance of TCD8^+^ cells between IRF8-high and IRF8-low groups were performed with Wilcoxon signed-rank test.

## Results

### Loss of IRF8 expression correlates with disease progression in BC

In order to investigate whether loss of IRF8 expression in BC is an important factor in disease progression and metastasis, we evaluated its expression by immunodetection of the protein in invasive ductal BC of various grades and sentinel lymph node metastases. Although information regarding the ER status of these samples was not available, our own analysis using Kaplan-Meier plotter data base show that most ER-negative tumors fall also into the more aggressive grade 3 phenotype (see Fig. 3a).

**Fig. 1:**
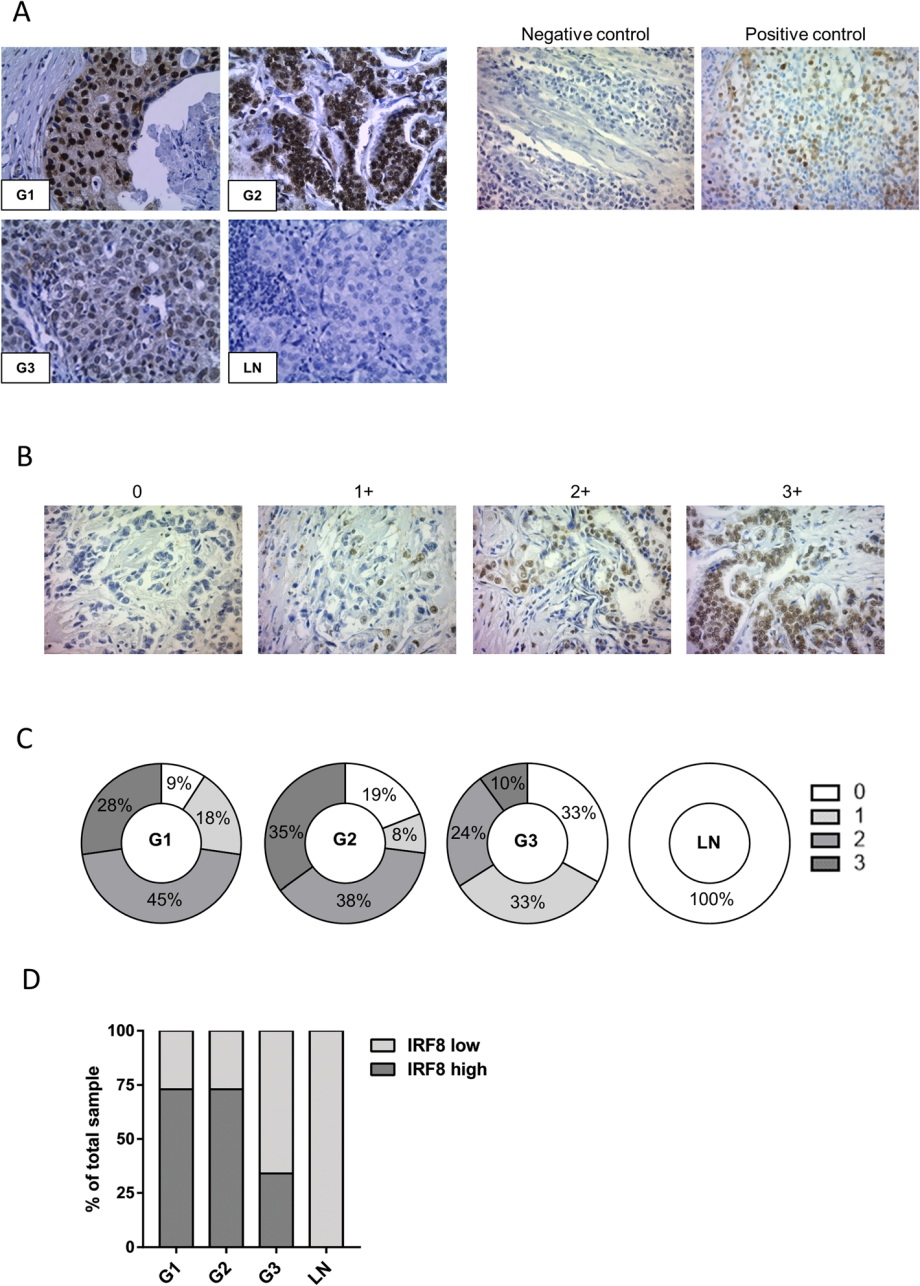
Immunohistochemical staining of IRF8 expression in BC and sentinel nodes samples. **a** Grade 1 breast tumors (G1, *n* = 11), grade 2 breast tumors (G2, *n* = 52), grade 3 breast tumors (G3, *n* = 21), and lymph node metastases (LN, *n* = 13) (left panel). Negative (secondary antibody alone) and positive controls (tonsils) are shown (right panel). Anti-IRF8 immunoreactivity is shown as the brown-stained cells, whereas cells that are unreactive to the anti-IRF8 antibody are indicated by the blue (hematoxylin) counterstain. Magnification, × 400. **b** Representative pictures of the different staining patterns for the entire score range from 0 to 3 (upper panel). Stratification of tumors according to staining intensity (score) for IRF8 (0: negative, + 1: weak, + 2: moderate, + 3: strong) (lower panel). **c** Stratification of the tumors as low or high IRF8 expression according to score classification (low: < 2 score, high: ≥ 2). *p* value as determined by Fisher’s exact test

Even if IRF8 is mainly expressed in the nuclei of tumor cells, weak IRF8 expression in cytoplasm was also detectable (Fig. 1a). Interestingly, IRF8 expression was robust in grade 1 and 2 tumors (G1 and G2, well- or moderately differentiated respectively) (Fig. 1a, b), showing a score ≥ 2+ in 73% of the samples analyzed (Fig. 1c). In contrast, only 34% of the grade 3 (G3) or poorly differentiated tumor samples exhibited high IRF8 expression (Fig. 1b, c). Furthermore, expression of IRF8 was not detected in metastatic foci in sentinel nodes (Fig. 1b, c). Also, the percentage of samples with high IRF8 expression diminished significantly as the disease worsens (*p* < 0.0001) and none of the invaded sentinel nodes evidenced expression of IRF8 (Fig. 1c).

**Fig. 2:**
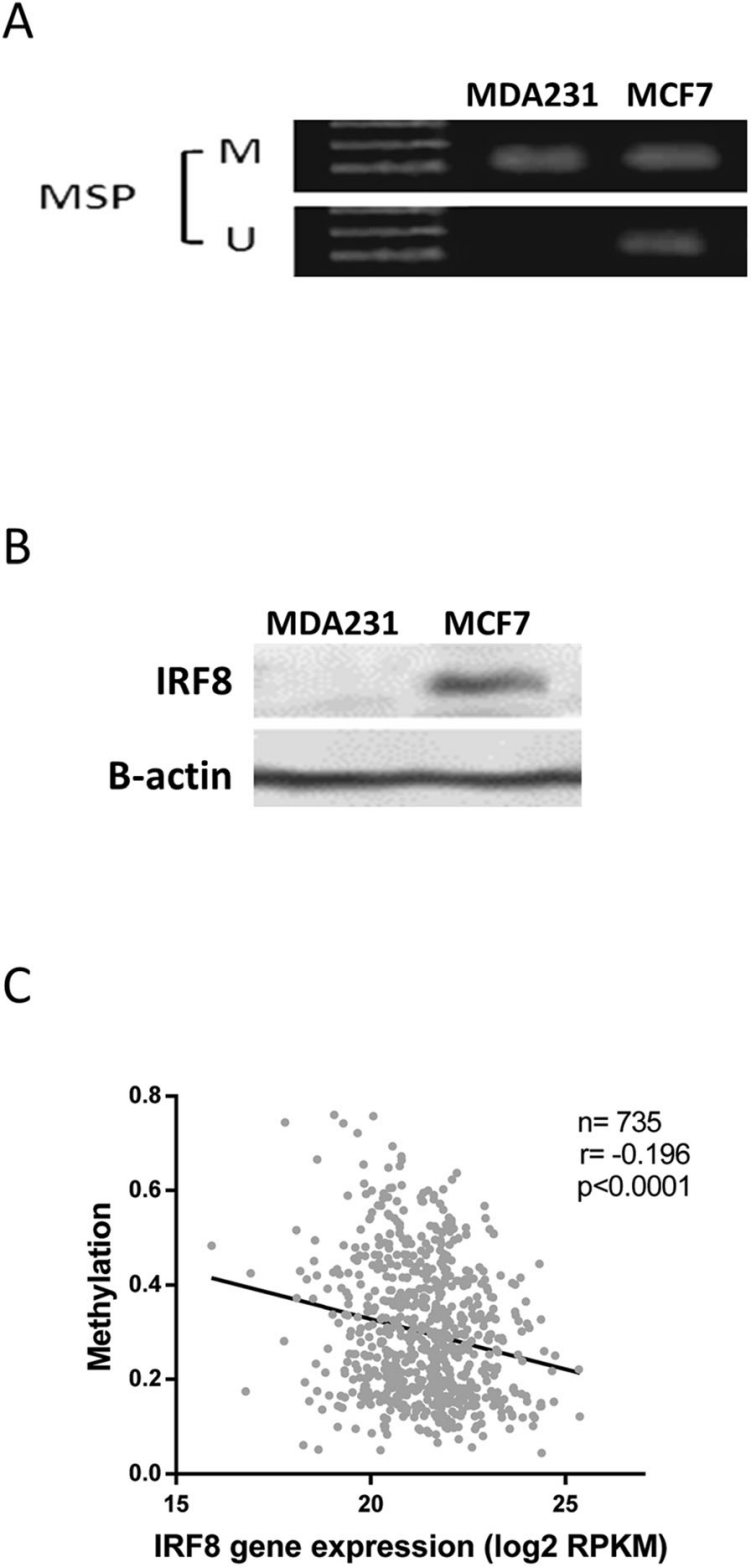
Methylation of IRF8 in BC cell lines and samples. **a** Methylation of the IRF8 promoter was evaluated by methylation-specific PCR (MSP) in the BC cell lines MDA231 and MCF7 (M: methylated; U: unmethylated). **b** Expression of IRF8 evaluated by western blot in MDA231 and MCF7 cells. **c** Correlation of IRF8 promoter methylation and gene expression in BC samples. Data are from The MethHC database tool (http://methhc.mbc.nctu.edu.tw/). Gene expression value was obtained from RNA Seq RPKM (reads per kilobase per million mapped reads) values in TCGA Data Portal. Data shown in **a** and **b** are representative of two experiments performed

Hypermethylation of IRF8 promoter has been reported to underlie IRF8 silencing or downregulation in multiple cancers, including BC [7, 9–11]. We therefore determined IRF8 promoter methylation status by a methylation-specific PCR test in the BC cell lines MCF7 and MDA231, which have low and high metastatic potential, respectively. As expected, the IRF8 promoter was readily methylated in MDA231 cells, accompanied by no expression of IRF8 (Fig. 2a, b), whereas the less aggressive MCF7 cell line expressed IRF8 and demonstrated simultaneous unmethylated and methylated promoter alleles, potentially indicating absence of homozygous silencing of IRF8 promoter in these cells. These results are in concordance with those observed by immunostaining where IRF8 expression is absent in MDA231 cells [10] and in BC samples with more aggressive phenotype [17]. To further verify the correlation of IRF8 methylation and its expression in BC samples, we took advantage of the MethHC database tool [20]. This exercise revealed that IRF8 promoter methylation correlates inversely with its gene expression (Fig. 2c), indicating that epigenetic changes are a major component of IRF8 downregulation. Altogether, our data indicate a potential prognostic value for IRF8 in BC progression, justifying a deeper characterization of its expression in different BC subtypes.

### IRF8 is a prognostic biomarker and predicts response to specific therapeutic regimens in ER-negative BC patients

**Fig. 3:**
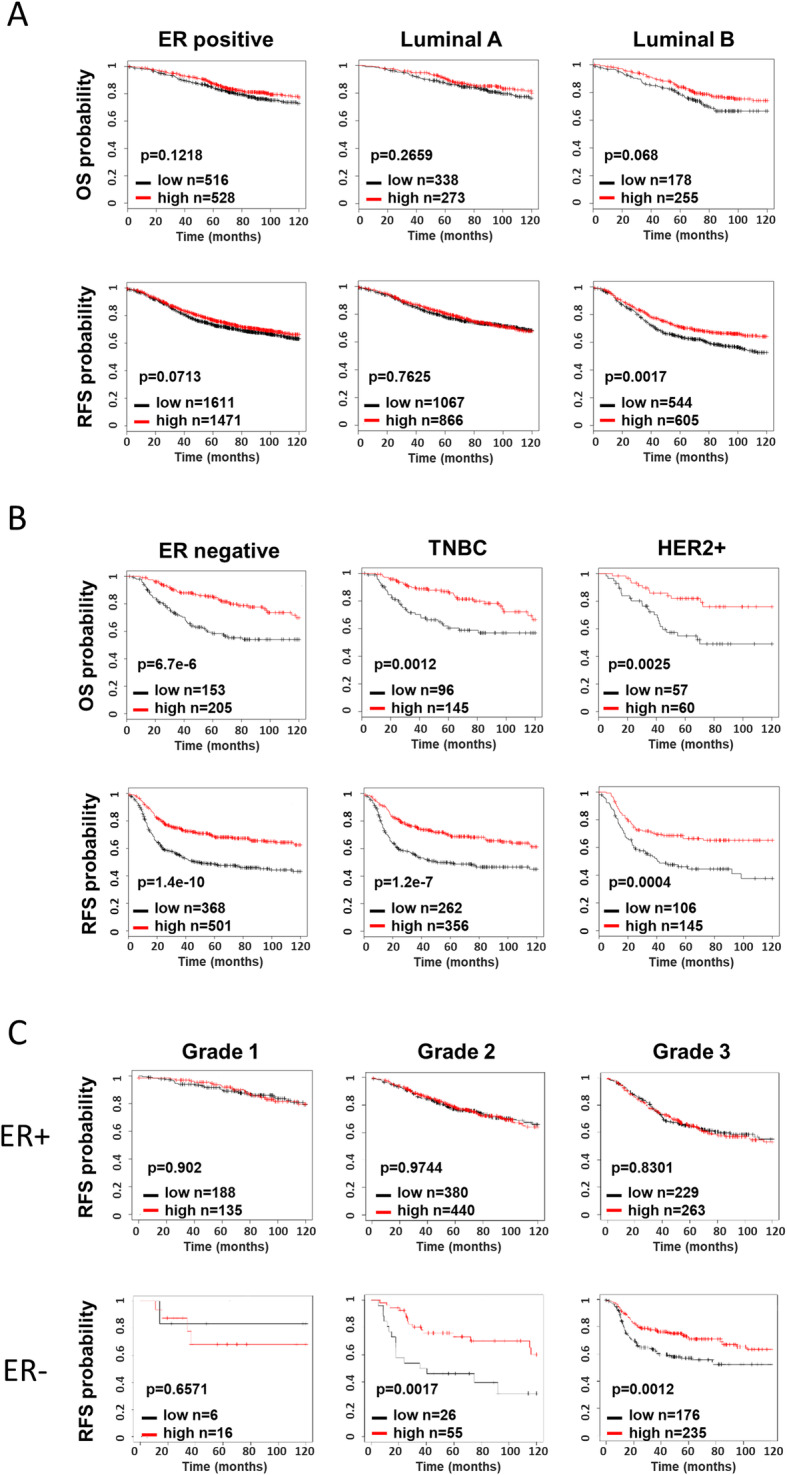
IRF8 is a prognostic marker in breast cancer ER-negative cancer. Kaplan-Meier plots of overall survival (OS) and relapse-free survival (RFS) by IRF8 status within molecular subtype BC. IRF8 groups were stratified using median cutoff of IRF8 value. The relationship between IRF8 expression and BC patients OS (**a**) and RFS (**b**) was analyzed using the Kaplan-Meier plotter data base (http://kmplot.com/analysis/). **c**) RFS was compared between high- and low-IRF8-expressing breast tumors for grade 1, grade 2, and grade 3 tumors. ER+: G1 (*n* = 323), G2 (*n* = 820), G3 (*n* = 492); ER−: G1 (*n* = 22), G2 (*n* = 81), G3 (*n* = 411). Patient samples were grouped based on the expression of IRF8 using the median cutoff value

To determine whether IRF8 could act as a biomarker that accurately stratifies patients for prognosis and potential response to therapies, we investigated whether tumor IRF8 expression correlated with improved outcome in distinct BC molecular subtypes. The online available database Kaplan–Meier plotter [22] was used to identify the relationship between IRF8 expression and overall survival or relapse-free survival. High expression of IRF8 was significantly associated with a longer overall and relapse-free survival in BC, but only in the ER-negative molecular subtypes, HER2+ and TNBC (Fig. 3a, b). Interestingly, most ER-negative subtypes are also grade 3 tumors. Indeed, according to KMplotter data base only 30% of the patients have ER+ grade 3 tumors in contrast to nearly 80% of the ER-negative patients which show grade 3 tumors. This has also been reported by Putti et al., 2005 [24]. Moreover, IRF8 expression predicts a better outcome only in the subgroup of ER-negative patients with grade 3 and 2 tumors (Fig. 3c), suggesting that IRF8 expression could more accurately discriminate the prognosis of ER-negative patients than the mere be histological classification.

To date, expression of the hormone receptor ER stratified patients for use of aromatase inhibitors or anti-estrogen therapy (tamoxifen), whereas expression of HER2+ indicates potential usefulness of trastuzumab. Chemotherapy is widely used as a combination of available choices according to the subtype of BC and clinical staging. The combination of fluorouracil, adriamycin, and cyclophosphamide (FAC) is a chemotherapy regimen sometimes given for localized BC with a relatively high risk for recurrence, whereas cyclophosphamide, methotrexate, and fluorouracil (CMF) is often used for earlier-stage BC that has not spread beyond the breast or lymph nodes. Thus, we determined if there was an association between IRF8 expression and the pathological complete response to these different therapeutic regimes.

**Fig. 4:**
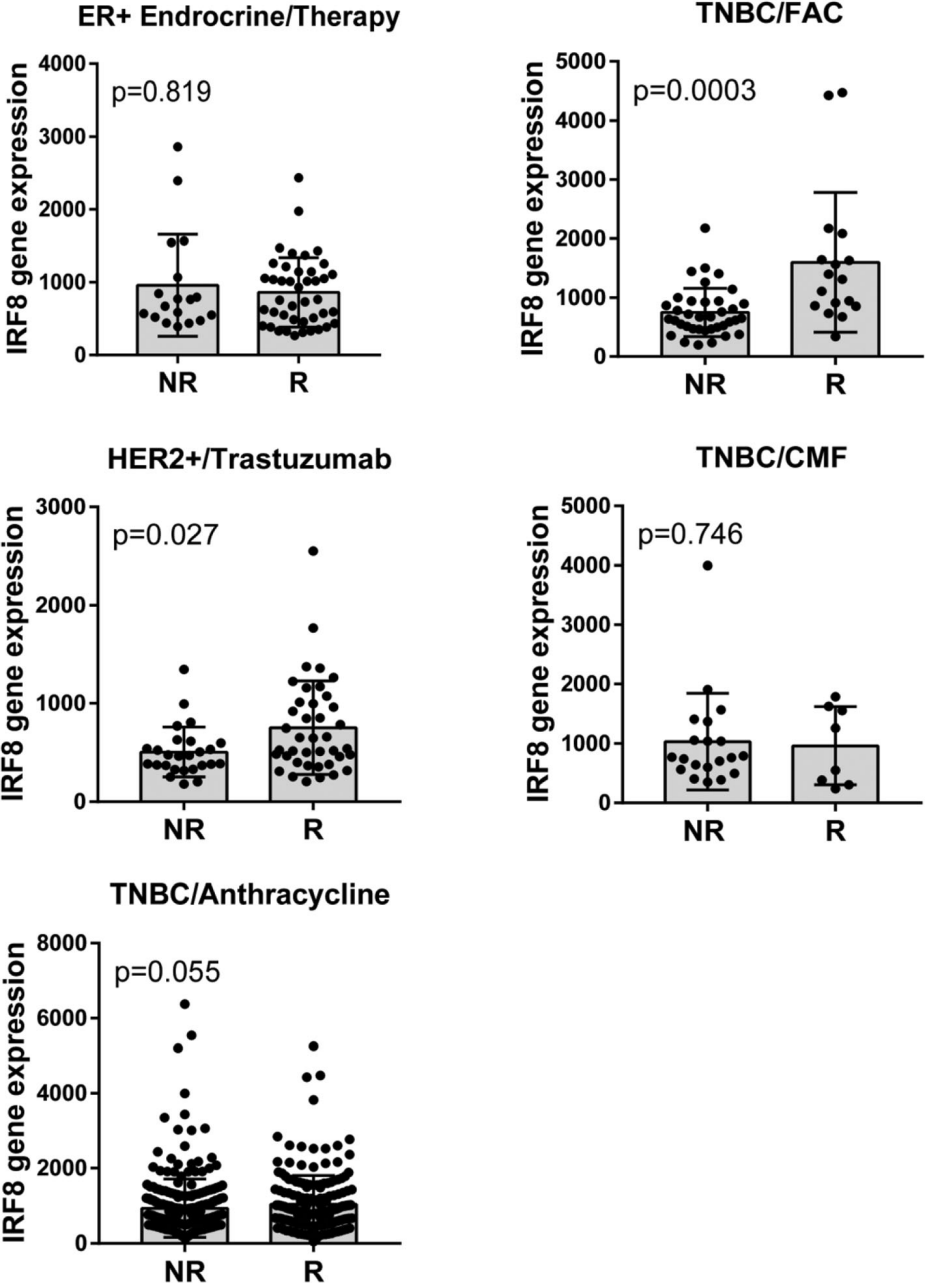
IRF8 is a predictive marker for complete pathological response to trastuzumab and FAC treatment in HER2+ and TNBC. IRF8 expression in non-responder (NR) and responder (R) patients to the different therapeutic regimes. ER+ BC: endocrine therapy treatment (*n* = 60), TNBC: FAC (*n* = 54), CMF (*n* = 28) or anthracycline (*n* = 473) treatments, and HER2+ BC: trastuzumab treatment (*n* = 66). Endocrine therapy involves: tamoxifen and aromatase inhibitors. FAC: fluorouracil, adriamycin (doxorubicin), and cytoxan (cyclophosphamide). CMF: cyclophosphamide, methotrexate, and fluorouracil

As anticipated, there was no significant association between IRF8 expression levels and response to endocrine therapy in patients with ER+ BC (luminal cancers). In contrast, in ER-negative BC (TNBC or HER2+), high expression levels of IRF8 was significantly associated with complete pathological response in patients treated with FAC (*p* = 0.0003) or trastuzumab (*p* = 0.027) but not CMF (*p* = 0.746) (Fig. 4). Interestingly, in TNBC, a higher expression of IRF8 was observed in patients who respond to anthracycline therapy, although not statistically different (*p* = 0.055). ROC curves confirmed the value of IRF8 as a prognostic factor of therapeutic response (Additional file 1: Figure S1).

In sum, these analyses indicate that IRF8 expression should be considered a prognostic biomarker in ER-negative BC where its expression also predicts complete pathological response to certain therapeutic regimes used for patients harboring these BC subtypes.

### Expression of IRF8 is associated with higher CD8^+^ T cell infiltration in BC

**Fig. 5:**
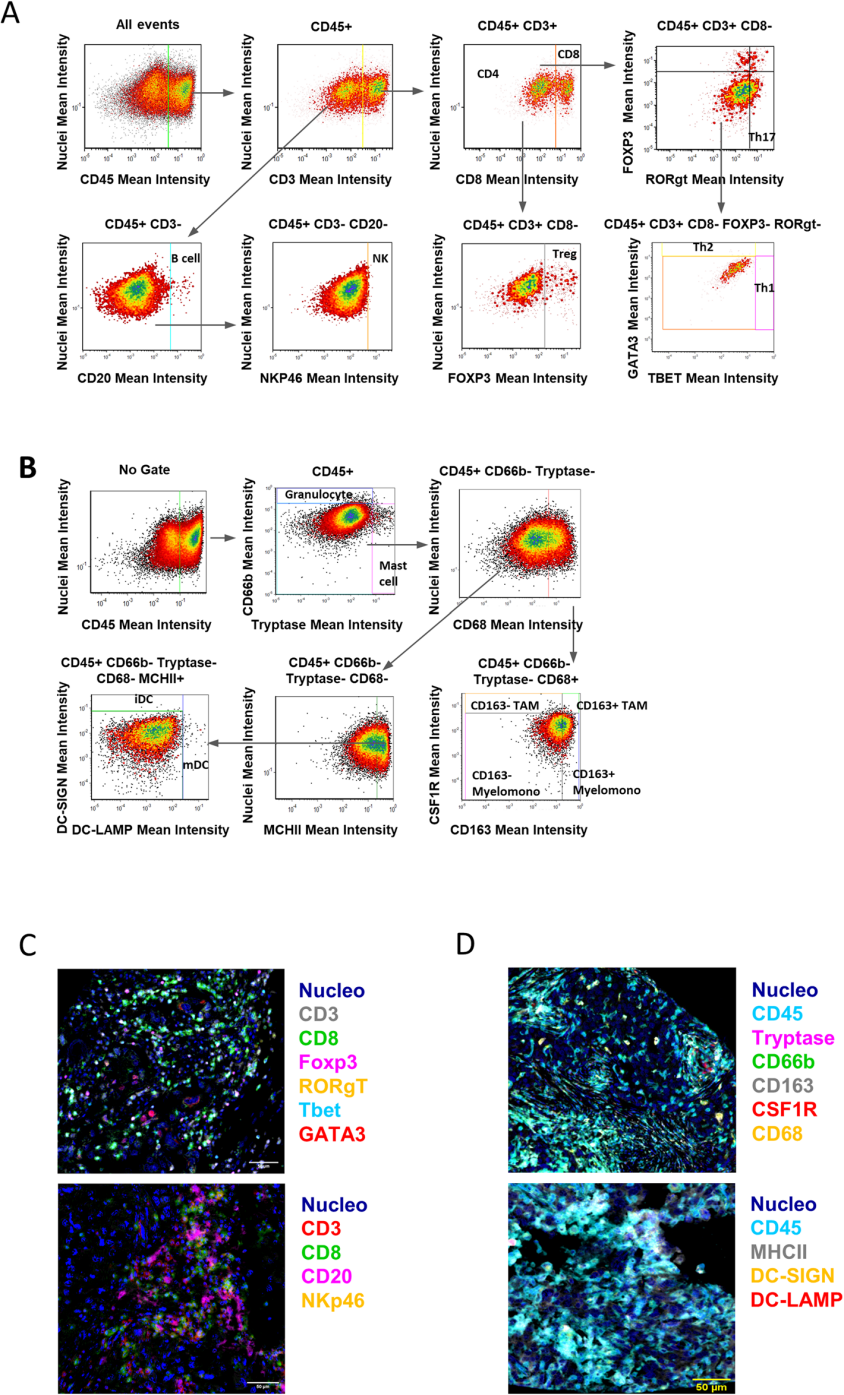
Multiparameter cytometric image analysis for quantification of multiplex IHC. Image cytometry-based cell population analyses for the lymphoid and myeloid biomarker panels are shown in **a** and **b**, respectively. Gating thresholds for qualitative identification were determined based on data in negative controls. TMA of BC samples were stained with the lymphoid (**c**) and myeloid (**d**) biomarker panels by pseudo-coloring. Scale bars, 50 μm

**Fig. 6:**
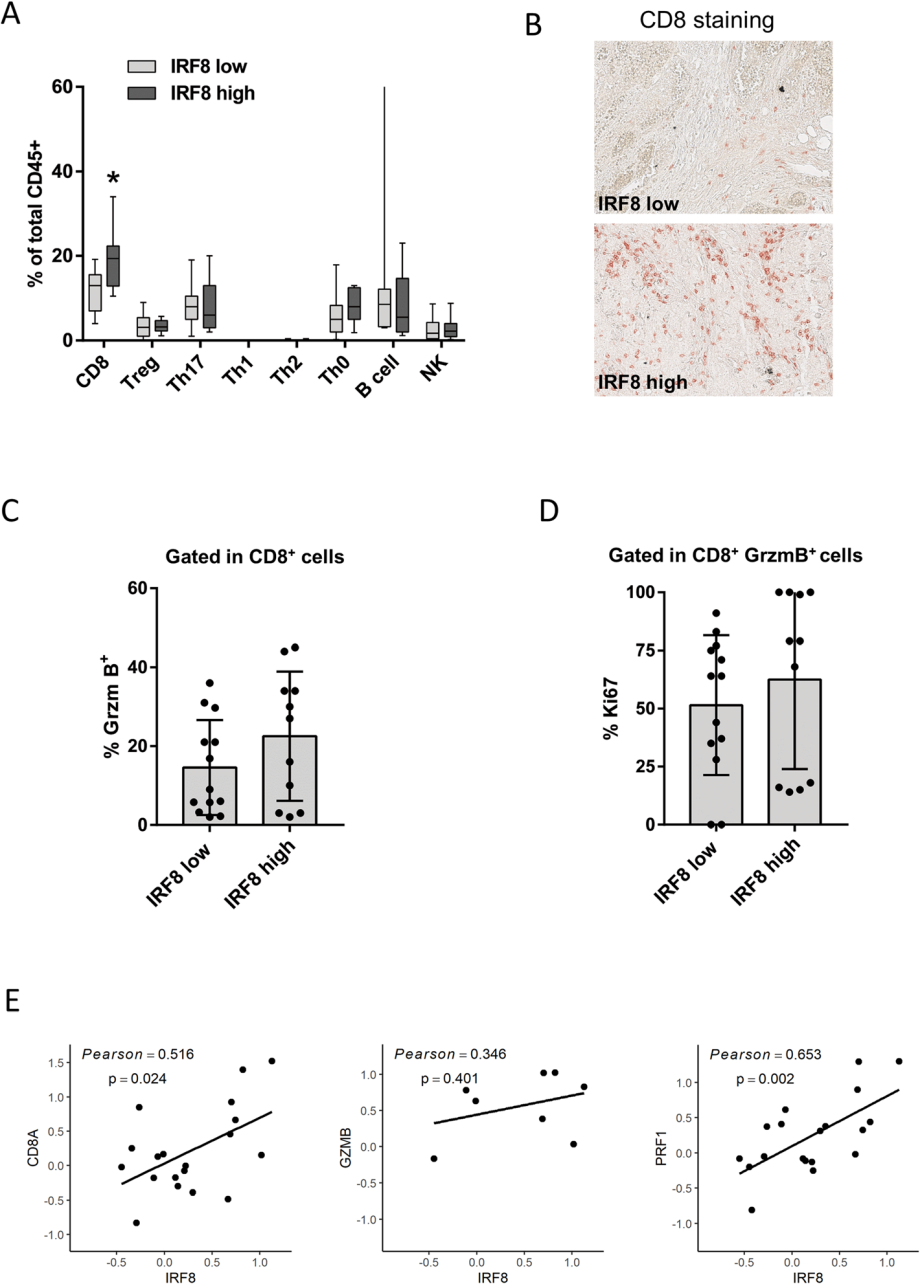
Higher cancer cell IRF8 expression levels is associated with an increased infiltration of CD8^+^ T cells. **a** Lymphoid cell percentages were quantified as a percentage of total CD45^+^ cells in ER-negative BC samples classified according to IRF8 cancer cell expression as in Fig. 1. **b** Representative images of CD8 staining in ER-negative BC by using quantitative multiplex immunohistochemistry (AEC chromogenic staining). **c** Percentage of CD8^+^GrzmB^+^ cells in the total CD8^+^ T cell population infiltrating tumors. **d** Percentage of Ki67^+^ in CD8^+^GrzmB^+^ T cell population. **e** Correlation between IRF8 protein expression and molecules associated with the antitumor immune response. *p < 0.05

Since high IRF8 expression levels are correlated with improved outcome in HER2+ and TNBC subtypes, we evaluated whether tumor cell IRF8 expression correlated with the complexity or contexture of tumor-infiltrating immune cells. To this end, we took advantage of a homemade TMA consisting of 24 ER-negative BC specimens, classified according to either low or high IRF8 cancer cell expression as determined by traditional immunohistochemistry. On the TMA, we performed sequential immunostaining of FFPE slides with two panels of 12 antibodies reporting leukocyte lineage for quantitative evaluation of lymphoid and myeloid lineage cells. Infiltrates were quantified by evaluating the chromogenic intensity of each cell using single-cell segmentation algorithms and image cytometry-based cell population analyses, an analytic process described previously [18]. A lineage gating strategy was used to identify each cell subpopulation (Fig. 5a, b). Chromogenic signals for lymphoid and myeloid biomarkers were then pseudo-colored and visualized in the tissue context (Fig. 5c, d). This analysis revealed a significant increase in the frequency of CD3^+^CD8^+^ T cells in tumors that also exhibited high neoplastic cell IRF8 expression (*p* < 0.05), whereas no differences were observed in the percentages of other lymphoid populations including Th0, Th1, Th2, Th17 T cells, regulatory T cells (Treg), CD20^+^ B cells, or natural killer (NK) cells (Fig. 6a, b). Interestingly, when we evaluated the differentiation and/or functional status of CD3^+^CD8^+^T cells, a higher percentage of these in tumors co-expressed granzyme B (GrzmB) correlating with high expression of IRF8 as compared to those with low expression of IRF8 (Fig. 6c), although it was not significantly different. In addition, the same tendency was seen in CD8^+^GrzmB^+^ cells, which also exhibited a more proliferative phenotype (Ki67-positivity) in tumors also expressing high levels of IRF8 (Fig. 6d), revealing that higher CD8^+^T cell infiltration was associated with a more activated phenotype. These data were also corroborated using iTRAQ-based mass spectrometry data from TCGA; this analysis allowed interrogation of protein expression in primary BC samples obtained from TCGA. Indeed, we determined a significant positive correlation between IRF8 protein expression and molecules associated with antitumor immune responses (e.g., CD8A, GZMB, PRF1) in ER-negative tumors (Fig. 6e). Moreover, when we used TCGA data combined with MIXTURE, an immune tumor microenvironment estimation method based on gene expression data, we validated again that IRF8 expression was associated with a relatively more abundant CD8^+^ T cell infiltration in ER-negative, HER2+, and TNBC subtypes (Additional file 2: Figure S2).

**Fig. 7:**
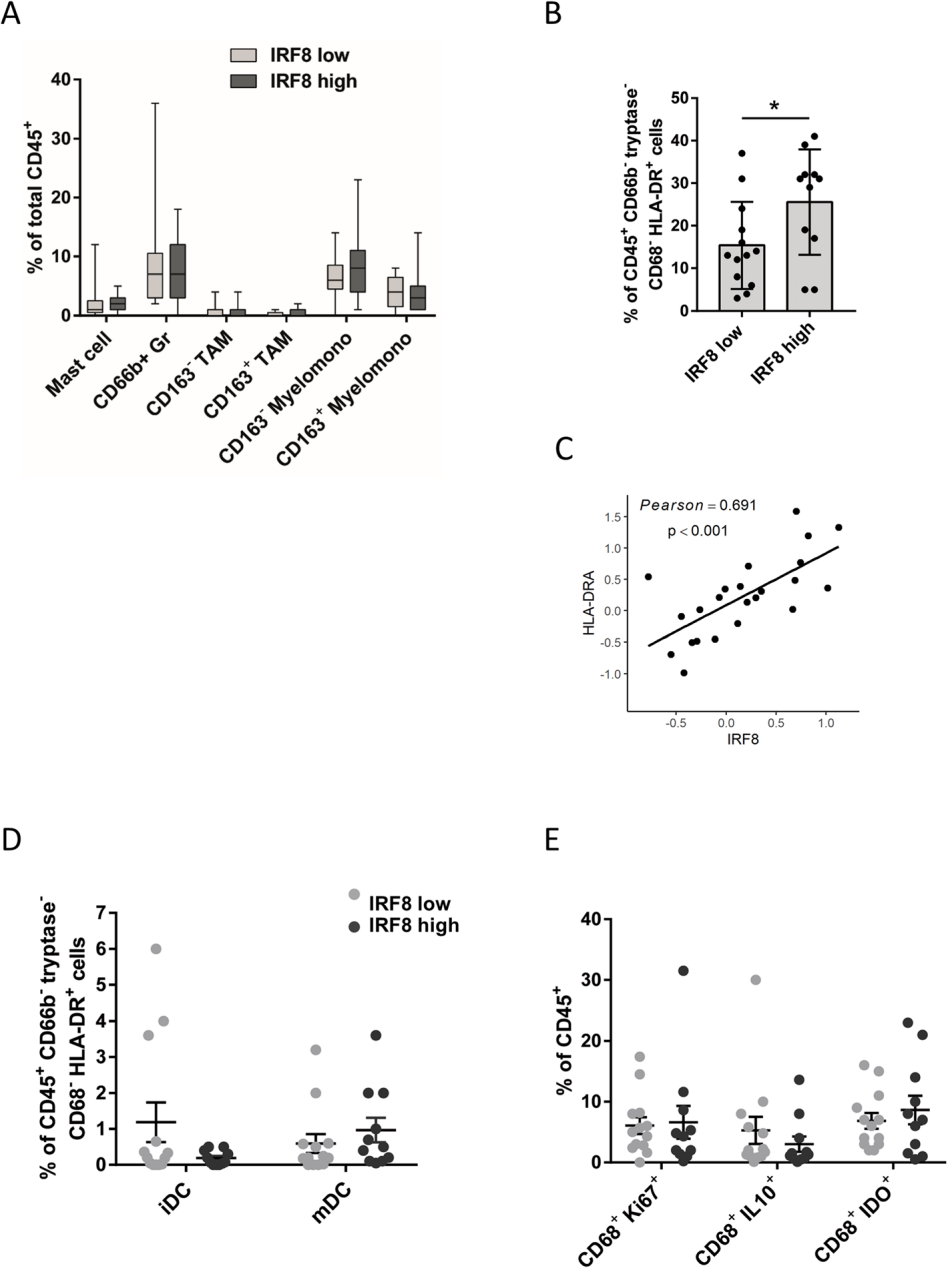
Higher cancer cell IRF8 expression levels is associated with an increased infiltration of CD45^+^ HLA-DR^+^ cells. **a** Myeloid cell percentages were quantified as a percentage of total CD45^+^ cells. **b** MHC II expression on CD45^+^ CD66b^−^ tryptase^−^ CD68^−^ cells. **c** Correlation between IRF8 protein expression and HLA-DRA evaluated using iTRAQ. **d** Immature (DCsign^+^) dendritic cells (iDC) and mature (LAMP^+^) dendritic cells (mDC) percentages were quantified as a percentage of CD45^+^ CD66b^−^ tryptase^−^ CD68^−^ HLA-DR^+^ cells. **e** Frequency of macrophages expressing Ki67, IL10, or IDO. *p < 0.05

With regard to myeloid cell infiltration of tumors, we did not observe significant differences in myeloid subsets correlating with IRF8 status (Fig. 7a), although a higher percentage of HLA-DR-expressing CD45^+^ cells were present in tumors with higher expression of IRF8 (*p* < 0.05) (Fig. 7b). Again, this result was corroborated using iTRAQ analysis, where we observed a positive correlation between IRF8 and HLA-DR expression (Fig. 7c). Interestingly, a non-significant decrease in immature dendritic cells, identified by the presence of the marker DC-sign, and IL-10-expressing macrophages (known to inhibit DC maturation via IL-10-dependent mechanisms [25]), was observed within tumors with high expression of IRF8 (Fig. 7d, e). Altogether, these results indicate that the presence of IRF8 in ER-negative BC is associated with an immune infiltration consistent with a more robust antitumor immune response.

## Discussion

Breast cancer is the leading cause of death from cancer in women worldwide [26]. When breast cancer is detected in early stages, treatment efficacy is superior resulting in improved clinical outcomes. However, if the diagnosis occurs in advanced stages or the disease, and depending on the molecular characteristics of the tumor, prognosis is usually less encouraging. The molecular characterization of breast cancers through determination of biomarkers (e.g., ER, PR, HER2, Ki67) has been useful both as a predictive metric, but also for designing therapeutic course [27–29]. Moreover, assessment of tumor-infiltrating lymphocytes (TILs) has an important prognostic role in TNBC [30, 31] and in HER2+ disease [32, 33]; however, patients stratified according to any of these attributes experience a wide range of clinical outcomes, supporting the need to identify and validate additional biomarkers easily evaluated in clinical labs and that could add precision in distinguishing clinical progression and therapeutic response of each patient.

In this study, our results support the proposition that IRF8 expression should be considered as a potent biomarker in ER-negative BC patients (TNBC and HER2+ patients). We examined expression of the IRF8 transcription factor at the protein level via immunohistochemistry where we validated, results obtained by in silico gene expression analysis, demonstrating that the percentage of tumors with high neoplastic cell IRF8 expression diminishes significantly as disease progresses. Moreover, none of the invaded sentinel nodes exhibited expression of nuclear IRF8. Our data corroborate previous results obtained by others reporting that IRF8 expression correlates inversely with IRF8 promoter methylation [7, 10, 11, 17], indicating that epigenetic changes are likely a major cause of IRF8 downregulation in BC cells and impact disease progression and metastasis. However, among ER-negative grade 3 tumors, there is a small subgroup which still has high IRF8 expression and consequently presents a better outcome. All together, these data indicate that IRF8 can be detected in some cases without epigenetic therapy and that the methylation silencing does not occur in all samples. Curiously, we found that high expression of IRF8 correlated with longer overall and relapse-free survival only in TN and HER2+ tumors, demonstrating that IRF8 is a prognostic marker for patients harboring ER-negative tumors.

In ER+ tumor samples, the presence of IRF8 appears to have no effect in discriminating the patient outcome, indicating that in this subgroup of patients, which bear mostly grade 1–2 tumors, there are other features or factors that are leading the tumor growth and that could act as better prognostic factors. In contrast, when ER signaling is absent, expression of this transcription factor becomes important, probably as a tumor suppressor gene, with impact in OS and RFS. Indeed, Luo et al. [17] showed that IRF8 performed as a candidate tumor suppressor by inducing G2/M phase cell cycle arrest and apoptosis in MDA-MB-231 (ER-) and T47D cells (ER+), and also by inhibiting cell migration and invasion in MDA-MB-231, but not in T47D cells. The effects of IRF8 thus seemed to be more pronounced in ER-negative breast cancer cells, supporting its prognostic value in this particular subtype.

Moreover, our analysis revealed that IRF8 expression predicts complete pathological response to trastuzumab therapy in HER2+ tumors, as well as to FAC chemotherapy in TNBC. It is known that anthracyclines stimulate rapid production of type I interferon (IFN-I) by malignant cells after activation of innate immune receptors by endogenous molecules released by dying tumor cells [34]. Thus, FAC therapy depends on endogenous IFN-I for success and consequently requires IRF8 expression for IFN-I induction as it binds to the promoters of IFN-I genes and participates in subsequent IRF7-mediated amplifying phase of IFN transcription [35]. These data explain why patients having low expression of IRF8 also exhibit a compromised response to FAC chemotherapeutic regimens and why success of this therapeutic approach relies on IRF8 levels. Likewise, Perez and colleagues reported a profile of 14 genes encoding various immune functions, which included IRF8, that were associated with a significantly improved relapse-free survival in patients with HER2+ BC treated with trastuzumab [36], supporting our data assigning IRF8 a predictive role to successful response to trastuzumab. Our findings are in line with several additional reports demonstrating that an IFN-I related signature predicts clinical response to anthracycline-based chemotherapy in several independent cohorts of patients with BC [34, 37]. Cyclophosphamide also impacts induction of antitumor immunity in vivo: it promotes expansion of CD8a^+^ DCs through induction of endogenous IFN-I and induces immunogenic tumor cell death, stimulating tumor infiltration and the engulfment of apoptotic material by DC to cross-prime CD8^+^ T cells [38]. Furthermore, sustained activation of the IFN-β/IFNAR/IRF7 signaling axis in chemotherapy-treated ER-negative BC cells instigates a state in which tumor cells are likely controlled by immune-mediated mechanisms [39].

Altogether, these data indicate that detecting IRF8 presence as a predictive factor by a simple immunohistochemistry assay could be a useful tool to be considered in clinical practice. On the other hand, presence of CD8^+^ cytotoxic T cells has been associated with better clinical outcomes in patients with HER2+ and TNBC [40, 41]. Expression of cancer-testis antigens such as MAGE-A and NY-ESO-1 are preferentially expressed in TNBC or high-grade and ER-negative BC [42–44], and their presence has been correlated with high levels of CD8^+^ TILs [45]. Our data indicate that a higher abundance of activated and effector CD8^+^ T cells are infiltrating tumors with higher expression of IRF8 in ER-negative BC samples. We hypothesize that patients expressing higher levels of IRF8 could present sustained levels of IFN-I within tumors, which would be needed to maintain fitness of the antitumor immune response, and consequently improved disease outcome. In colon epithelial cells, IRF8 has been shown to regulate the expression levels of osteopontin which is a potent suppressor of CD8+ T cell response and consequently proposed to be a new checkpoint. IRF8 binds to the ISRE elements at the *Spp1* promoter and represses its expression. Osteopontin levels are elevated in human colon cancer patient periphery, correlating with decreased disease-specific survival [46]. A similar mechanism could be involved in BC patients. However, a fraction of TN and ER-negative tumors that were included in the study herein fell in the more aggressive grade 3 category which, according to data, silence IRF8 expression by epigenetic mechanisms, although at the same time, are heavily infiltrated. Thus, outcome of these patients likely reflects a balance between the lack of the tumor suppressor functions of IRF8 and the antitumor immune response, which in these tumors could arise partly from the elevated expression of tumor-associated antigens. Along these lines, IRF8 functions extrinsically as a GM-CSF repressor in T cells [47]. Loss of IRF8 function increases secretion of both G-CSF and GM-CSF, ligands that upon high affinity cognate receptor activation on myeloid progenitor cells promote myeloid-mediated T cell suppression, in alignment with the inverse relationship between IRF8 levels and these cells observed in patients with BC and coinciding with poorer prognosis [48].

## Conclusion

Our study demonstrates that high levels of IRF8 represent a favorable prognostic indicator in ER-negative (HER2+ and TNBC) breast cancer. Moreover, IRF8 expression distinguishes between responders and non-responders to specific therapies in HER2+ and TNBC, BC subtypes that present a relatively high risk for recurrence. Our results indicate that a strong correlation exists between activated and effector CD8^+^ T cell infiltration and neoplastic cell IRF8 expression and together support the proposition that IRF8 should be considered as a clinically relevant biomarker for therapeutic decisions. Moreover, these findings support the need for ongoing studies to identify and develop therapeutic approaches to maintain intratumoral levels of IRF8 in order to maintain immunological microenvironments thwarting tumor progression.

## Supplementary Information

**Additional file 1: Figure S1**. ROC curves of IRF8 validated for FAC and trastuzumab treatment in TNBC and HER2+ tumors. ER+: endocrine therapy treatment (*n* = 60), TNBC: FAC (*n* = 54), CMF (*n* = 28), or anthracycline (*n* = 473) regimens treatment, and HER2+: Trastuzumab treatment (*n* = 66). Endocrine therapy: tamoxifen and aromatase inhibitor. FAC: fluorouracil, adriamycin (doxorubicin) and cytoxan (cyclophosphamide). CMF: cyclophosphamide, methotrexate and fluorouracil.

**Additional file 2: Figure S2.** CD8 relative abundance in IRF8^hi^ and IRF8^lo^ tumors. TCGA data base and MIXTURE analysis showed higher relative abundance of CD8 infiltration in ER-negative, HER2+ and TN tumor with high IRF8 expression. Samples were grouped on the basis of the expression of IRF8 using the median cutoff value.

## Data Availability

Not applicable

## Abbreviations

IRF8: Interferon regulatory factor 8
CRC: Colorectal carcinoma (CRC)
TCGA: The Cancer Genome Atlas
MDSC: Myeloid derived-suppressor cells
IFN-γ: Interferon gamma
BC: Breast cancer
ER: Estrogen receptor
HER2: Human epidermal growth factor receptor 2
TNBC: Triple-negative breast cancer
TMA: Tissue microarray
FFPE: Formalin-fixed and paraffin embedded
MS-PCR: Methylation-specific PCR
iTRAQ: Isobaric tags for relative and absolute quantification
GrzmB: Granzyme B
TILs: Tumor-infiltrating lymphocytes
IFN-I: Type I interferon

## References

[1] Feng J, Wang H, Shin DM, Masiuk M, Qi CF, Morse HC (2011). IFN regulatory factor 8 restricts the size of the marginal zone and follicular B cell pools. J Immunol.

[2] Holtschke T, Löhler J, Kanno Y, Fehr T, Giese N, Rosenbauer F, Lou J, Knobeloch KP, Gabriele L, Waring JF, Bachmann MF, Zinkernagel RM, Morse HC, Ozato K, Horak I (1996). Immunodeficiency and chronic myelogenous leukemia-like syndrome in mice with a targeted mutation of the ICSBP gene. Cell..

[3] Kim SH, Burton J, Yu CR, Sun L, He C, Wang H, Morse HC, Egwuagu CE (2015). Dual-function of the IRF8 transcription factor in autoimmune uveitis: loss of IRF8 in T cells exacerbates uveitis while Irf8 deletion in the retina confers protection. J Immunol.

[4] Durai V, Bagadia P, Granja JM, Satpathy AT, Kulkarni DH, Davidson JT, Wu R, Patel SJ, Iwata A, Liu TT (2019). Cryptic activation of an Irf8 enhancer governs cDC1 fate specification. Nat Immunol.

[5] Bagadia P, Huang X, Liu TT, Durai V, Grajales-Reyes GE, Nitschké M, Modrusan Z, Granja JM, Satpathy AT, Briseño CG, Gargaro M, Iwata A, Kim S, Chang HY, Shaw AS, Murphy TL, Murphy KM (2019). An Nfil3-Zeb2-Id2 pathway imposes Irf8 enhancer switching during cDC1 development. Nat Immunol.

[6] Karki R, Lee E, Place D, Samir P, Mavuluri J, Sharma BR, Balakrishnan A, Malireddi RKS, Geiger R, Zhu Q (2018). IRF8 regulates transcription of Naips for NLRC4 inflammasome activation. Cell.

[7] Yang D, Thangaraju M, Greeneltch K, Browning DD, Schoenlein PV, Tamura T, Ozato K, Ganapathy V, Abrams SI, Liu K (2007). Repression of IFN regulatory factor 8 by DNA methylation is a molecular determinant of apoptotic resistance and metastatic phenotype in metastatic tumor cells. Cancer Res.

[8] Yan M, Wang H, Sun J, Liao W, Li P, Zhu Y, Xu C, Joo J, Sun Y, Abbasi S, Kovalchuk A, Lv N, Leonard WJ, Morse HC (2016). Cutting Edge: expression of IRF8 in gastric epithelial cells confers protective innate immunity against Helicobacter pylori infection. J Immunol.

[9] Ibrahim ML, Klement JD, Lu C, Redd PS, Xiao W, Yang D, Browning DD, Savage NM, Buckhaults PJ, Morse HC (2018). Myeloid-derived suppressor cells produce IL-10 to elicit DNMT3b-dependent IRF8 silencing to promote colitis-associated colon tumorigenesis. Cell Rep.

[10] Lee KY, Geng H, Ng KM, Yu J, van Hasselt A, Cao Y, Zeng YX, Wong AH, Wang X, Ying J (2008). Epigenetic disruption of interferon-gamma response through silencing the tumor suppressor interferon regulatory factor 8 in nasopharyngeal, esophageal and multiple other carcinomas. Oncogene..

[11] Suzuki M, Ikeda K, Shiraishi K, Eguchi A, Mori T, Yoshimoto K, Shibata H, Ito T, Baba Y, Baba H (2014). Aberrant methylation and silencing of IRF8 expression in non-small cell lung cancer. Oncol Lett.

[12] Liang J, Lu F, Li B, Liu L, Zeng G, Zhou Q, Chen L (2019). IRF8 induces senescence of lung cancer cells to exert its tumor suppressive function. Cell Cycle.

[13] Abrams SI (2010). A multi-functional role of interferon regulatory factor-8 in solid tumor and myeloid cell biology. Immunol Res.

[14] Hu X, Yang D, Zimmerman M, Liu F, Yang J, Kannan S, Burchert A, Szulc Z, Bielawska A, Ozato K, Bhalla K, Liu K (2011). IRF8 regulates acid ceramidase expression to mediate apoptosis and suppresses myelogeneous leukemia. Cancer Res.

[15] Ye L, Xiang T, Zhu J, Li D, Shao Q, Peng W, Tang J, Li L, Ren G (2018). Interferon consensus sequence-binding protein 8, a tumor suppressor, suppresses tumor growth and invasion of non-small cell lung cancer by interacting with the Wnt/β-catenin pathway. Cell Physiol Biochem.

[16] Graff-Baker AN, Orozco JIJ, Marzese DM, Salomon MP, Hoon DSB, Goldfarb M (2018). Epigenomic and transcriptomic characterization of secondary breast cancers. Ann Surg Oncol.

[17] Luo X, Xiong X, Shao Q, Xiang T, Li L, Yin X, Li X, Tao Q, Ren G (2017). The tumor suppressor interferon regulatory factor 8 inhibits β-catenin signaling in breast cancers, but is frequently silenced by promoter methylation. Oncotarget..

[18] Gatti G, Quintar AA, Andreani V, Nicola JP, Maldonado CA, Masini-Repiso AM, Rivero VE, Maccioni M (2009). Expression of Toll-like receptor 4 in the prostate gland and its association with the severity of prostate cancer. Prostate..

[19] Tsujikawa T, Kumar S, Borkar RN, Azimi V, Thibault G, Chang YH, Balter A, Kawashima R, Choe G, Sauer D, el Rassi E, Clayburgh DR, Kulesz-Martin MF, Lutz ER, Zheng L, Jaffee EM, Leyshock P, Margolin AA, Mori M, Gray JW, Flint PW, Coussens LM (2017). Quantitative multiplex immunohistochemistry reveals myeloid-inflamed tumor-immune complexity associated with poor prognosis. Cell Rep.

[20] Schneider CA, Rasband WS, Eliceiri KW (2012). NIH image to ImageJ: 25 years of image analysis. Nat Methods.

[21] Huang WY, Hsu SD, Huang HY, Sun YM, Chou CH, Weng SL, Huang HD (2015). MethHC: a database of DNA methylation and gene expression in human cancer. Nucleic Acids Res.

[22] Gyorffy B, Lanczky A, Eklund AC, Denkert C, Budczies J, Li Q, Szallasi Z (2010). An online survival analysis tool to rapidly assess the effect of 22,277 genes on breast cancer prognosis using microarray data of 1809 patients. Breast Cancer Res Treatment.

[23] Fekete JT, Győrffy B (2019). ROCplot.org: validating predictive biomarkers of chemotherapy/hormonal therapy/anti-HER2 therapy using transcriptomic data of 3,104 breast cancer patients. Int J Cancer.

[24] Putti TC, El-Rehim DM, Rakha EA, Paish CE, Lee AH, Pinder SE, Ellis IO (2005). Estrogen receptor-negative breast carcinomas: a review of morphology and immunophenotypical analysis. Mod Pathol.

[25] Ruffell B, Chang-Strachan D, Chan V, Rosenbusch A, Ho CM, Pryer N, Daniel D, Hwang ES, Rugo HS, Coussens LM (2014). Macrophage IL-10 blocks CD8+ T cell-dependent responses to chemotherapy by suppressing IL-12 expression in intratumoral dendritic cells. Cancer Cell.

[26] Bertucci F, Houlgatte R, Benziane A, Granjeaud S, Adélaïde J, Tagett R, Loriod B, Jacquemier J, Viens P, Jordan B (2000). Gene expression profiling of primary breast carcinomas using arrays of candidate genes. Hum Mol Genet.

[27] Cheang MC, Chia SK, Voduc D, Gao D, Leung S, Snider J, Watson M, Davies S, Bernard PS, Parker JS (2009). Ki67 index, HER2 status, and prognosis of patients with luminal B breast cancer. J Natl Cancer Inst.

[28] Desmedt C, Haibe-Kains B, Wirapati P, Buyse M, Larsimont D, Bontempi G, Delorenzi M, Piccart M, Sotiriou C (2008). Biological processes associated with breast cancer clinical outcome depend on the molecular subtypes. Clin Cancer Res.

[29] Dai X, Li T, Bai Z, Yang Y, Liu X, Zhan J, Shi B (2015). Breast cancer intrinsic subtype classification, clinical use and future trends. Am J Cancer Res.

[30] Salgado R, Denkert C, Demaria S, Sirtaine N, Klauschen F, Pruneri G, Wienert S, Van den Eynden G, Baehner FL, Penault-Llorca F, Perez EA (2015). The evaluation of tumor-infiltrating lymphocytes (TILs) in breast cancer: recommendations by an International TILs Working Group 2014. Ann Oncol.

[31] Loi S, Drubay D, Adams S, Pruneri G, Francis PA, Lacroix-Triki M, Joensuu H, Dieci MV, Badve S, Demaria S, Gray R, Munzone E, Lemonnier J, Sotiriou C, Piccart MJ, Kellokumpu-Lehtinen PL, Vingiani A, Gray K, Andre F, Denkert C, Salgado R, Michiels S (2019). Tumor-infiltrating lymphocytes and prognosis: a pooled individual patient analysis of early stage triple-negative breast cancers. J Clin Oncol.

[32] Dieci MV, Conte P, Bisagni G, Brandes AA, Frassoldati A, Cavanna L, Musolino A, Giotta F, Rimanti A, Garrone O, Bertone E, Cagossi K, Sarti S, Ferro A, Piacentini F, Maiorana A, Orvieto E, Sanders M, Miglietta F, Balduzzi S, D’Amico R, Guarneri V (2019). Association of tumor-infiltrating lymphocytes with distant disease-free survival in the Short HER randomized adjuvant trial for patients with early HER2+ breast cancer. Ann Oncol.

[33] Denkert C, von Minckwitz G, Darb-Esfahani S, Lederer B, Heppner BI, Weber KE, Budczies J, Huober J, Klauschen F, Furlanetto J, Schmitt WD, Blohmer JU, Karn T, Pfitzner BM, Kümmel S, Engels K, Schneeweiss A, Hartmann A, Noske A, Fasching PA, Jackisch C, van Mackelenbergh M, Sinn P, Schem C, Hanusch C, Untch M, Loibl S (2018). Tumour-infiltrating lymphocytes and prognosis in different subtypes of breast cancer: a pooled analysis of 3771 patients treated with neoadjuvant therapy. Lancet Oncol.

[34] Sistigu A, Yamazaki T, Vacchelli E, Chaba K, Enot DP, Adam J, Vitale I, Goubar A, Baracco EE, Remédios C, Fend L, Hannani D, Aymeric L, Ma Y, Niso-Santano M, Kepp O, Schultze JL, Tüting T, Belardelli F, Bracci L, la Sorsa V, Ziccheddu G, Sestili P, Urbani F, Delorenzi M, Lacroix-Triki M, Quidville V, Conforti R, Spano JP, Pusztai L, Poirier-Colame V, Delaloge S, Penault-Llorca F, Ladoire S, Arnould L, Cyrta J, Dessoliers MC, Eggermont A, Bianchi ME, Pittet M, Engblom C, Pfirschke C, Préville X, Uzè G, Schreiber RD, Chow MT, Smyth MJ, Proietti E, André F, Kroemer G, Zitvogel L (2014). Cancer cell-autonomous contribution of type I interferon signaling to the efficacy of chemotherapy. Nat Med.

[35] Tailor P, Tamura T, Kong HJ, Kubota T, Kubota M, Borghi P, Gabriele L, Ozato K (2007). The feedback phase of type I interferon induction in dendritic cells requires interferon regulatory factor 8. Immunity..

[36] Perez EA, Thompson EA, Ballman KV, Anderson SK, Asmann YW, Kalari KR, Eckel-Passow JE, Dueck AC, Tenner KS, Jen J, Fan JB, Geiger XJ, McCullough AE, Chen B, Jenkins RB, Sledge GW, Winer EP, Gralow JR, Reinholz MM (2015). Genomic analysis reveals that immune function genes are strongly linked to clinical outcome in the North Central Cancer Treatment Group N9831 adjuvant trastuzumab trial. J Clin Oncol.

[37] Brockwell NK, Rautela J, Owen KL, Gearing LJ, Deb S, Harvey K, Spurling A, Zanker D, Chan CL, Cumming HE, Deng N, Zakhour JM, Duivenvoorden HM, Robinson T, Harris M, White M, Fox J, Ooi C, Kumar B, Thomson J, Potasz N, Swarbrick A, Hertzog PJ, Molloy TJ, Toole SO’, Ganju V, Parker BS (2019). Tumor inherent interferon regulators as biomarkers of long-term chemotherapeutic response in TNBC. NPJ Precis Oncol.

[38] Schiavoni G, Sistigu A, Valentini M, Mattei F, Sestili P, Spadaro F, Sanchez M, Lorenzi S, D'Urso MT, Belardelli F, Gabriele L, Proietti E, Bracci L (2011). Cyclophosphamide synergizes with type I interferons through systemic dendritic cell reactivation and induction of immunogenic tumor apoptosis. Cancer Res.

[39] Lan Q, Peyvandi S, Duffey N, Huang YT, Barras D, Held W, Richard F, Delorenzi M, Sotiriou C, Desmedt C, Lorusso G, Rüegg C (2019). Type I interferon/IRF7 axis instigates chemotherapy-induced immunological dormancy in breast cancer. Oncogene..

[40] Kroemer G, Senovilla L, Galluzzi L, André F, Zitvogel L (2015). Natural and therapy-induced immunosurveillance in breast cancer. Nat Med.

[41] Stanton SE, Disis ML (2016). Clinical significance of tumor-infiltrating lymphocytes in breast cancer. J Immunother Cancer.

[42] Dushyanthen S, Beavis PA, Savas P, Teo ZL, Zhou C, Mansour M, Darcy PK, Loi S (2015). Relevance of tumor-infiltrating lymphocytes in breast cancer. BMC Med.

[43] Chen YT, Ross DS, Chiu R, Zhou XK, Chen YY, Lee P, Hoda SA, Simpson AJ, Old LJ, Caballero O, Neville AM (2011). Multiple cancer/testis antigens are preferentially expressed in hormone-receptor negative and high-grade breast cancers. PLoS One.

[44] Curigliano G, Viale G, Ghioni M, Jungbluth AA, Bagnardi V, Spagnoli GC, Neville AM, Nolè F, Rotmensz N, Goldhirsch A (2011). Cancer-testis antigen expression in triple-negative breast cancer. Ann Oncol.

[45] Ademuyiwa FO, Bshara W, Attwood K, Morrison C, Edge SB, Karpf AR, James SA, Ambrosone CB, O'Connor TL, Levine EG, Miliotto A, Ritter E, Ritter G, Gnjatic S, Odunsi K (2012). NY-ESO-1 cancer testis antigen demonstrates high immunogenicity in triple negative breast cancer. PLoS One.

[46] Klement JD, Paschall AV, Redd PS, Ibrahim ML, Lu C, Yang D, Celis E, Abrams SI, Ozato K, Liu K (2018). An osteopontin/CD44 immune checkpoint controls CD8+ T cell activation and tumor immune evasion. J Clin Invest.

[47] Paschall AV, Zhang R, Qi CF, Bardhan K, Peng L, Lu G, Yang J, Merad M, McGaha T, Zhou G, Mellor A, Abrams SI, Morse HC, Ozato K, Xiong H, Liu K (2015). IFN regulatory factor 8 represses GM-CSF expression in T cells to affect myeloid cell lineage differentiation. J Immunol.

[48] Waight JD, Netherby C, Hensen ML, Miller A, Hu Q, Liu S, Bogner PN, Farren MR, Lee KP, Liu K, Abrams SI (2013). Myeloid-derived suppressor cell development is regulated by a STAT/IRF-8 axis. J Clin Invest.

