# Correction to: High IRF8 expression correlates with CD8 T cell infiltration and is a predictive biomarker of therapy response in ER-negative breast cancer

**DOI:** 10.1186/s13058-021-01427-6

**Published:** 2021-04-22

**Authors:** Gerardo Gatti, Courtney Betts, Darío Rocha, Maribel Nicola, Verónica Grupe, Cecilia Ditada, Nicolas G. Nuñez, Emiliano Roselli, Paula Araya, Jeremías Dutto, Lucia Boffelli, Elmer Fernández, Lisa M. Coussens, Mariana Maccioni

**Affiliations:** 1 Laboratorio de Investigación en Cáncer, Fundación para el progreso de la Medicina, X5000EMS, Córdoba, Argentina; 2 Consejo Nacional de Investigaciones Científicas y Técnicas (CONICET), Buenos Aires, Argentina; 3 Department of Cell, Developmental & Cancer Biology, Knight Cancer Institute, Oregon Health & Science University, Portland, OR USA; 4 Facultad de Ciencias Exactas, Físicas y Naturales, Universidad Nacional de Córdoba, Córdoba, Argentina; 5 Institute of Experimental Immunology, University of Zurich, Zurich, Switzerland; 6 Departamento de Bioquímica Clínica, Centro de Investigaciones en Bioquímica Clínica e Inmunología (CIBICI), Consejo Nacional de Investigaciones Científicas y Técnicas (CONICET), Facultad de Ciencias Químicas, Universidad Nacional de Córdoba, 5000 Córdoba, Argentina; 7 CIDIE-CONICET, Universidad Católica de Córdoba, Córdoba, Argentina

**Correction to: Breast Cancer Res 23, 40 (2021)**

**https://doi.org/10.1186/s13058-021-01418-7**

After publication of the original article [1], the authors identified some errors in the authors’ affiliations list. The correct affiliations are below listed:

Lisa M Cussens (3)

3) Department of Cell, Developmental & Cancer Biology, Knight Cancer Institute, Oregon Health & Science University, Portland, Oregon, USA

Elmer Fernández (2, 7)

2) Consejo Nacional de Investigaciones Científicas y Técnicas (CONICET)

7) CIDIE-CONICET, Universidad Católica de Córdoba, Córdoba, Argentina

Mariana Maccioni (6)

6) Departamento de Bioquímica Clínica, Centro de Investigaciones en Bioquímica Clínica e Inmunología (CIBICI), Consejo Nacional de Investigaciones Científicas y Técnicas (CONICET), Facultad de Ciencias Químicas, Universidad Nacional de Córdoba, Córdoba 5000, Argentina

The original article has been corrected.
