# Supplementary figures and images for: High IRF8 expression correlates with CD8 T cell infiltration and is a predictive biomarker of therapy response in ER-negative breast cancer
Source: Breast Cancer Res. 2021 Mar 25;23:40. doi: 10.1186/s13058-021-01418-7 (PMC7992828; doi:10.1186/s13058-021-01418-7)

# Additional file 1: Figure S1

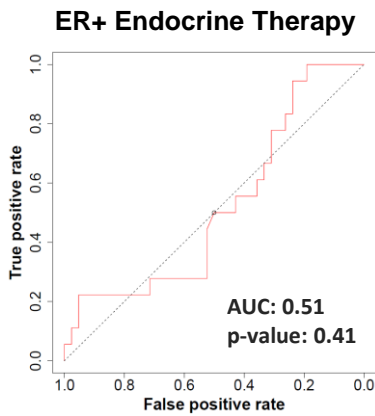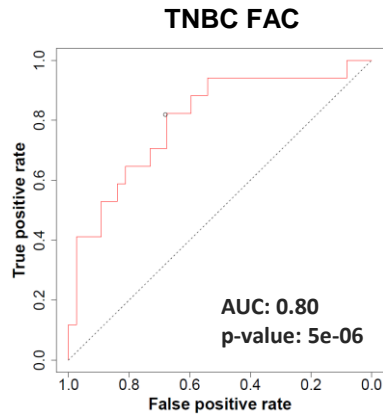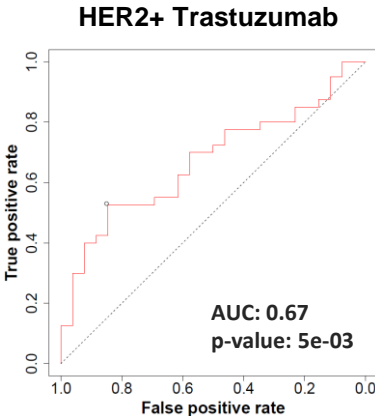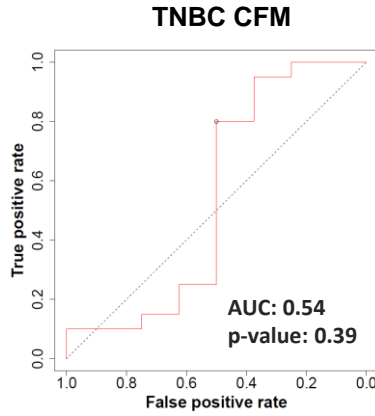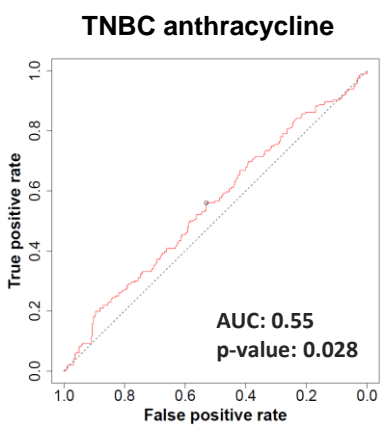

Supplement: Supplementary file 1 — Additional file 1: Figure S1. ROC curves of IRF8 validated for FAC and trastuzumab treatment in TNBC and HER2+ tumors. ER+: endocrine therapy treatment (n = 60), TNBC: FAC (n = 54), CMF (n = 28), or anthracycline (n = 473) regimens treatment, and HER2+: Trastuzumab treatment (n = 66). Endocrine therapy: tamoxifen and aromatase inhibitor. FAC: fluorouracil, adriamycin (doxorubicin) and cytoxan (cyclophosphamide). CMF: cyclophosphamide, methotrexate and fluorouracil. [file 13058_2021_1418_MOESM1_ESM.pdf]

Additional file 2: Figure S2

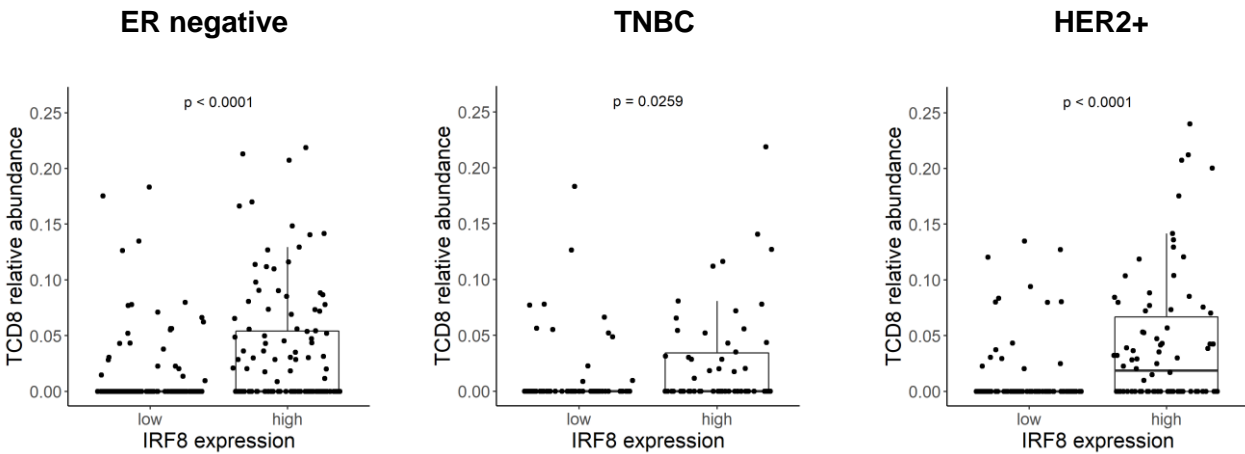

Supplement: Supplementary file 2 — Additional file 2: Figure S2. CD8 relative abundance in IRF8hi and IRF8lo tumors. TCGA data base and MIXTURE analysis showed higher relative abundance of CD8 infiltration in ER-negative, HER2+ and TN tumor with high IRF8 expression. Samples were grouped on the basis of the expression of IRF8 using the median cutoff value. [file 13058_2021_1418_MOESM2_ESM.pdf]
